# A realistic computational model for the formation of a Place Cell

**DOI:** 10.1038/s41598-023-48183-5

**Published:** 2023-12-08

**Authors:** Camille Mazzara, Michele Migliore

**Affiliations:** 1 Department of Promoting Health, Maternal-Infant. Excellence and Internal and Specialized Medicine (PROMISE) G. D’Alessandro, University of Palermo, Palermo, Italy; 2 Institute of Biophysics, National Research Council, Palermo, Italy

**Keywords:** Neuroscience, Mathematics and computing

## Abstract

Hippocampal Place Cells (PCs) are pyramidal neurons showing spatially localized firing when an animal gets into a specific area within an environment. Because of their obvious and clear relation with specific cognitive functions, Place Cells operations and modulations are intensely studied experimentally. However, although a lot of data have been gathered since their discovery, the cellular processes that interplay to turn a hippocampal pyramidal neuron into a Place Cell are still not completely understood. Here, we used a morphologically and biophysically detailed computational model of a CA1 pyramidal neuron to show how, and under which conditions, it can turn into a neuron coding for a specific cue location, through the self-organization of its synaptic inputs in response to external signals targeting different dendritic layers. Our results show that the model is consistent with experimental findings demonstrating PCs stability within the same spatial context over different trajectories, environment rotations, and place field remapping to adapt to changes in the environment. To date, this is the only biophysically and morphologically accurate cellular model of PCs formation, which can be directly used in physiologically accurate microcircuits and large-scale model networks to study cognitive functions and dysfunctions at cellular level.

## Introduction

A population of pyramidal CA1 neurons in the hippocampus, called Place Cells (PCs), are preferentially activated when an animal reaches a specific area in an environment. Directly linked with the ability of an animal to carry out a cognitive function, PCs have been intensely studied experimentally. It has been found that they act as an internal cognitive map^1,2^, ensuring correct spatial navigation together with other cells, such as head direction cells^3–5^, border cells^6,7^, grid cells^8^ and speed cells^9^. PCs have been identified in different mammals, e.g. in bats and primates^10–15^. However, although a large amount of data has been gathered, the cellular processes that may be involved, or need to interplay, to turn a hippocampal pyramidal neuron into a PC are not completely understood. In the CA1 region of the hippocampus, it has been shown that the interaction between an input from the Entorhinal Cortex with one from the Shaffer Collaterals (SCs) can induce the formation of a Place Cell^16^ through an exquisite interaction between these independent, spatially distributed, and well-timed signals. Unfortunately, their integration dynamics is practically impossible to investigate experimentally in vivo*,* and a more detailed picture is needed to better understand not only the basic mechanisms underlying PC formation, but also those cognitive processes directly depending on normal PCs operations. For these reasons, we developed and present here a realistic computational model of PC formation. Our results suggest how, to what extent, and under what conditions, dendritic integrations and synaptic plasticity processes can turn a CA1 pyramidal neuron into a Place Cell.

## Materials and methods

All simulations were carried out using the NEURON simulation environment (v7.5, Carnevale and Hines, 2006). At time of publication, all model and simulation files will be available on ModelDB (http://modeldb.yale.edu/267613), and as an interactive entry in the “live papers” section of the Cellular Level Simulation Platform of the EBRAINS Infrastructure (https://humanbrainproject.github.io/hbp-bsp-live-papers).

The neuron morphology and channel kinetics are the same as those previously published paper^17^ and systematically validated against several experimental findings on CA1 pyramidal neurons. The morphology is composed of a soma, axon, 58 basal dendrites, 67 membrane sections forming the main apical trunk and distal tuft, and 74 oblique dendrites (30 of which stemming out of the main apical trunk). We used Na^+^ and K_DR_ currents, two A-type potassium currents (K_A_, for proximal and distal dendrites), a non-specific I_h_ current, a M-type potassium current, K_M_. The K_A_ and I_h_ peak conductance increased linearly with distance from the soma^18–20^ and the K_M_ was inserted in the soma and axon.

Live animals or cell lines were not involved in this study.

### Model implementation

**Figure 1:**
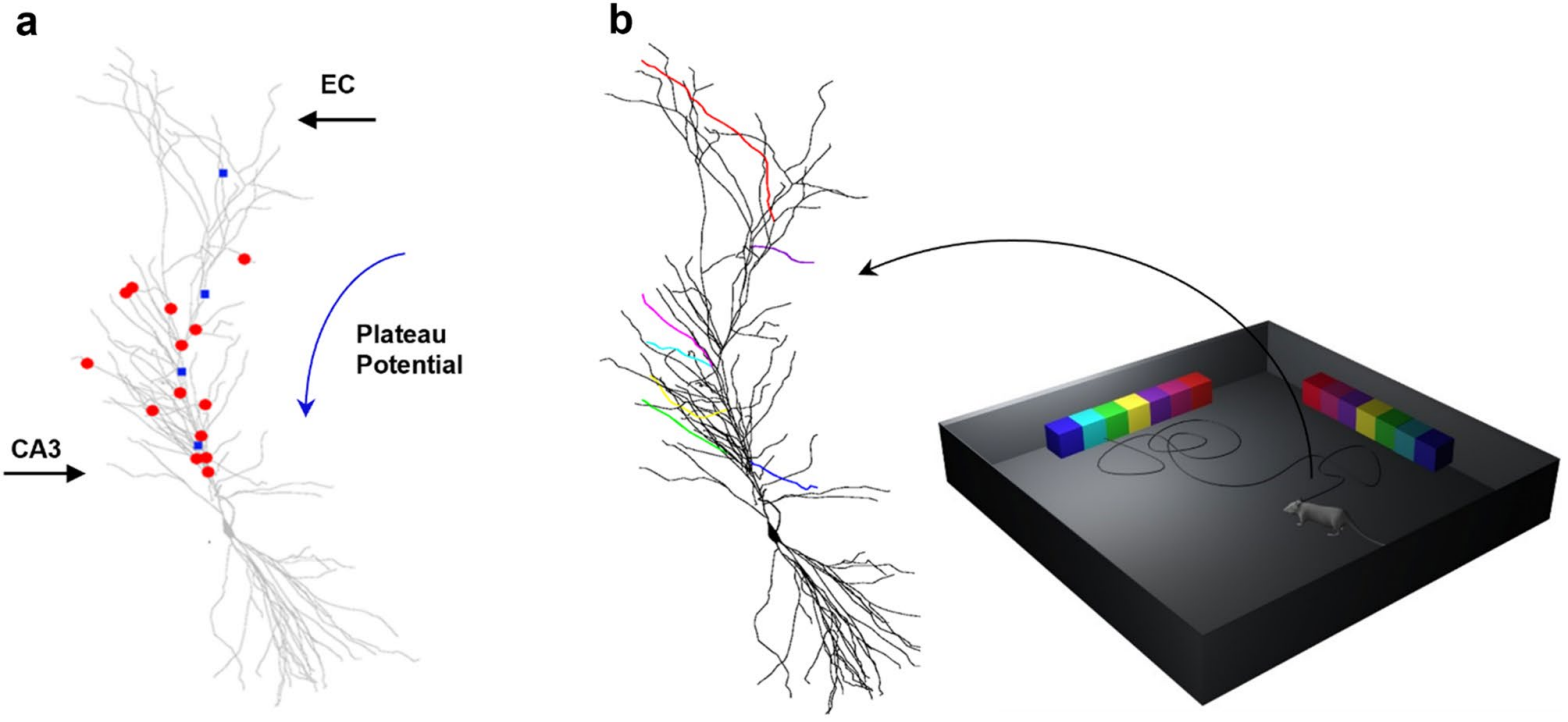
Schematic representation of the model. (**a**) A PC formation requires the interaction between a proximal input from the Shaffer Collateral (CA3) with a distal input from the entorhinal cortex (EC), strong enough to generate a dendritic plateau potential. The blue arrow indicates the propagation of the Plateau Potential after the EC activation; red circles represent SC synapses; blue squares represent the input current locations used to mimic the Plateau Potential propagation. (**b**) Schematic representation of the virtual environment used in all simulations; different features of an object (colored cubes) in the visual field activated different oblique dendrites; the spot light indicates the current visual field.

**Figure 2:**
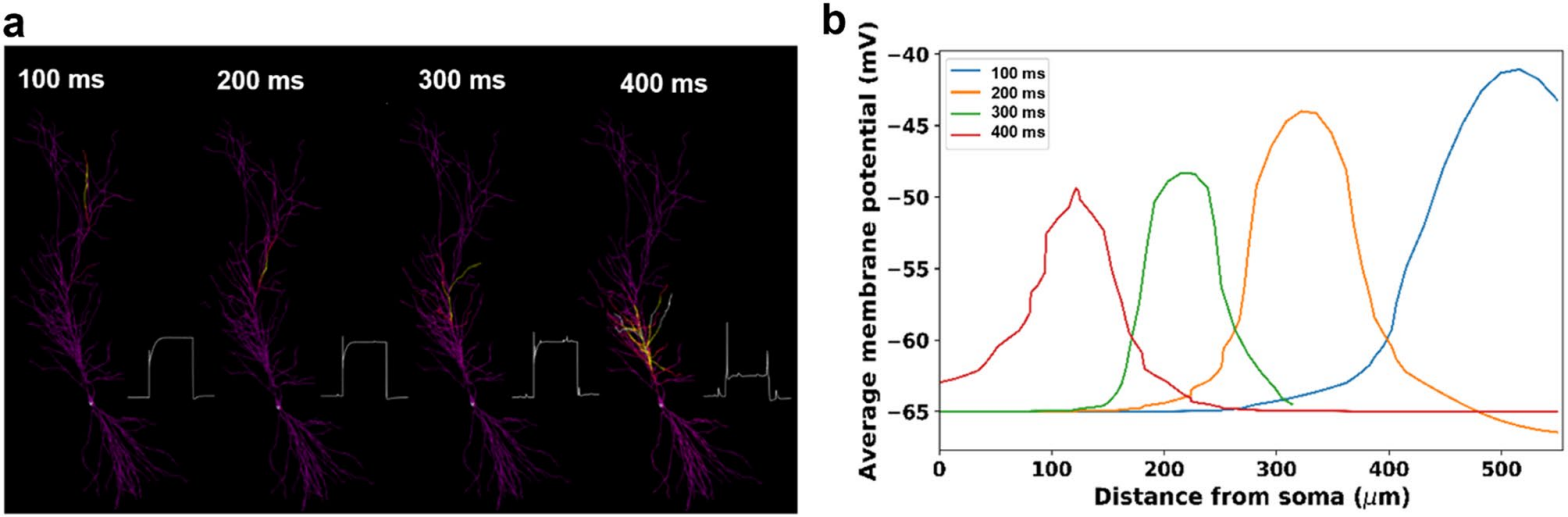
Implementation of the Plateau Potential. (**a**) Membrane depolarization at four instants during a typical simulation. Membrane potential is color coded, with more depolarized segments depicted in white/yellow; the insets represent the membrane voltage recorded during 300 ms time windows at the most depolarized dendritic locations of each snapshot; (**b**) Average membrane potential as a function of the distance from the soma at different times.

The implementation of the model and its operation are schematically illustrated in Fig. 1a; they were directly inspired by experimental findings suggesting that Place Cells can be formed by an appropriate binding of two dendritic signals^16^. In a CA1 pyramidal neuron two main spatially segregated excitatory inputs converge, representing highly preprocessed sensorial and contextual signals: (1) from the Perforant Pathway, originating directly from the Entorhinal Cortex and innervating the distal apical region (EC, in Fig. 1a), and (2) from the Schaeffer Collaterals, originating from the CA3 region and activating synapses onto the proximal oblique dendrites (CA3, in Fig. 1a). Experiments suggested that their interplay, while an animal explores an environment looking for a reward, could be the basic process through which a Place Cell is formed.

In our model, inputs from CA3 (schematically represented by red circles in Fig. 1a) were implemented with excitatory synapses composed of an AMPA and a NMDA component, modeled as in Gasparini et al.^21^. The AMPA component was implemented as a double-exponential conductance change, with 0.5 and 1 ms for the rise and decay time, respectively, and a reversal potential of 0 mV. The NMDA conductance was implemented following a kinetic scheme fitting experimental data^22^, with an external Mg^2+^ concentration of 1 mM and a reversal potential of 0 mV. Synapses were placed on oblique dendrites, consistently with experimental findings suggesting that most of the excitatory synaptic input on CA1 pyramidal cells target oblique dendrites^23^. To reduce possible artifacts caused by the specific dendritic morphology used in this work, the peak synaptic conductance in each oblique dendrite was independently adjusted to be just below the spiking threshold when activated at background frequency. Consistently with experimental findings^24^, this resulted in a local dendritic depolarization increasing with distance from the soma while generating essentially the same peak somatic depolarization. A supplementary figure (Fig. S1) has been included in the revision, showing typical EPSPs recorded in four different oblique dendrites, with the inset showing the average somatic depolarization in response to individual synaptic activation (light red area represents variance).

The effect of the EC activation was empirically implemented by sequentially activating four stimulating electrodes positioned along the main apical trunk of the neuron, as schematically shown in Fig. 1a (blue squares). The activation of the first electrode started the plateau potential in a distal dendritic location. This corresponds to the response to a relatively strong EC activity in the tuft. To mimic its forward propagation toward the soma, the four 200 ms long current injection steps at different apical trunk locations were sequentially activated every 500 ms, starting from the tuft location and continuing with the more proximal ones. The resulting membrane potential dynamics mimics the forward propagation of a physiologically complex and computationally much more expensive calcium wave. As long as the virtual animal stays in the same spatial location during the plateau propagation, the presynaptic inputs on the oblique dendrites, coding for the environmental cues, and the forward propagating postsynaptic depolarization will jointly create the conditions to potentiate all the involved synapses, eventually forming the Place Cell. It should be clear that the process is robust and able to work independently from the implementation details of the underlying processes, with the critical mechanism being the animal staying around the same spatial location during the plateau propagation.

We wanted to test whether a dendritic plateau potential, forward propagating toward the soma, can induce plasticity selectively in those synapses activated by environmental cues during spatial navigation, to form (or remap) a PC. In Fig. 1b, we show a conceptual representation of the model operation. During a simulation, a virtual animal explores a square arena containing different cues (rainbow objects in Fig. 1b), following a random trajectory. The field of vision of the animal is 90°, with decreasing resolution with increasing distance. Depending on the animal location and head direction, objects (or parts of them) can enter the visual field. The underlying assumption is that specific object’s features activate different dendrites, through different CA3 fibers, eliciting local dendritic action potentials that can eventually generate a somatic output to signal object recognition^25^. This is consistent with experimental findings in humans, suggesting that individual hippocampal neurons are selectively activated by impressively distinct pictures of individuals, landmarks, or objects^11,26,27^. In the model, we split an object in parts (colored boxes in Fig. 1b) and associated each part to a different oblique dendrite. In general, different objects activated different dendrites, according to the set of features they were composed of. The number of dendrites to use to code for an object is arbitrary in the absence of specific experimental evidence. During preliminary test simulations (not shown), we also successfully tested the coding of an object using 5 dendrites. We have finally chosen to follow modeling results^25^ suggesting that 7 (± 2) may be the most effective combination. A detailed investigation of how many dendrites need to be used, as well as their location in the tree, size, distance from soma, or intrinsic electrophysiological properties was out of the scope for this work. To code for two objects, we thus colocalized 14 AMPA + NMDA synapses on 14 different oblique dendrites, with each synapse modeling the synchronous activation of a group of synapses. The model thus does not consider the details of the nonlinear spatial and temporal dendritic input summation of a group of inputs. We are aware of the many processes modulating dendritic signal integration in CA1 pyramidal neurons^21^. However, as for the other strategic choices we took in this work, we wanted to balance computational efficiency and biophysical/morphological accuracy in such a way to be able to use hundreds of thousands of this type of single cell models into a full-scale rodent CA1 network implementation^28^,or even several millions for the full scale of a human CA1^29^. For this reason, we had to use an effective, instead of a detailed, implementation for many processes. In this case, we decided to lump all the synaptic mechanisms responsible for local dendritic spiking into computationally fast AMPA and NMDA synapses. Even without an intracellular calcium dynamics, which would not qualitatively change the results, the dendritic integration process of the model is still much more realistic than any of the spiking neuron implementations universally used to model large scale networks of brain regions.

Under control conditions, synapses not corresponding to any of the known features in the visual field were randomly (Poisson) activated at an average frequency of 3Hz (in the range of the theta rhythm). When known features (in our case parts of an object) were present in the visual field, the corresponding synapses (schematically represented by colored dendrites in Fig. 1b) were activated at an average frequency in the high gamma range (80 Hz). Different parts of an object entering the visual field independently activated different dendrites, generating local dendritic action potentials that eventually elicited a somatic action potential^25,30^. Synaptic activation rates were chosen following both experimental and theoretical results, suggesting that working memory is organized by oscillatory processes at theta and gamma^31^. Furthermore, a computational model^32^, based on experimental findings, suggested that the periodic reactivation (or replay) of information, coordinated by theta and gamma neural oscillations, facilitates working memory, and neuronal activity in the gamma range could play an important role in attention, memory tasks^33^ and in other cognitive functions^34^.

### Synaptic plasticity

Synaptic weights were updated following a Spike Time Dependent Plasticity (STDP) rule already used in previous work^35^, consistent with experimental findings in CA1 pyramidal neurons^36^. More specifically, the peak synaptic conductance of each synapse was updated at any occurrence of a pre- or post-synaptic spike time (*t*_*pre*_ or *t*_*post*_, respectively) as:

$$g_{peak} \left( {t + dt} \right) \, = \, g^{0}_{peak} + \, A\left( t \right)$$

$$\begin{aligned}A\left(t+dt\right) &= A\left(t\right)* \left(1-d*\frac{{{e}^{-\frac{\left(\left(t_{post}-t_{pre}\right)-M\right)}{2{V}^{2}}}}^{2}}{V\sqrt{2\pi }}\right) \\ & \quad \quad for (t_{post}- t_{pre}) < 0 \;\; and\end{aligned}$$

$$\begin{aligned} A(t+dt) & = A(t) * \left({g}_{peak}^{max}-{g}_{peak}^{0}-A\left(t\right)\right) *p* {e}^{-\frac{(t_{post}-t_{pre})}{\tau }} \\ & \quad \quad for (t_{post} - t_{pre}) > 0 \end{aligned}$$

where the parameters M = − 24, V = 6.32, τ = 2, d = 0.3, and p = 1 were chosen to be consistent with experimental findings^36^, ${\mathrm{g}}_{\mathrm{peak}}^{0}$ indicates the initial peak conductance and ${\mathrm{g}}_{\mathrm{peak}}^{\mathrm{max}}$ its maximum value. A dendritic spike time was calculated as the time in which the local membrane potential crossed the threshold of – 10 mV.

## Results

It has been suggested^16^ that one of the key mechanisms underlying the process of associating the two main excitatory inputs targeting a CA1 pyramidal neuron, appears to be a depolarizing plateau potential initiated in the distal apical tree and propagating toward the soma.

We empirically implemented this process by sequentially activating a series of current steps at four locations in the major apical trunk (see “Materials and methods”). In this implementation, where we are considering an isolated cell, the initial current step in a distal dendrite starts at a predetermined time for mere convenience. In a circuit, it will be up to the modeler to straightforwardly connect the plateau initiation with the most appropriate voltage threshold detector in a distal dendritic location. The overall result will not change. The activation of the current injections resulted in a depolarizing wave that progressively affected dendritic regions closer and closer to the soma, as suggested by experiments^16^. The process is illustrated in Fig. 2a, where we show four snapshots taken during a movie of a simulation (Suppl. Movie SM1) in which we activated the four electrodes in the absence of any background synaptic activity. The snapshots show the neuron membrane potential at four different times during the simulation, and the local dendritic membrane potential during the entire simulation. To better illustrate the signal’s propagation, in Fig. 2b we show the average membrane potential as a function of the distance from the soma, calculated at different time instants during the simulation. In the following paragraphs, we will show how this mechanism can play a determinant role in Place Cell formation.

**Figure 3:**
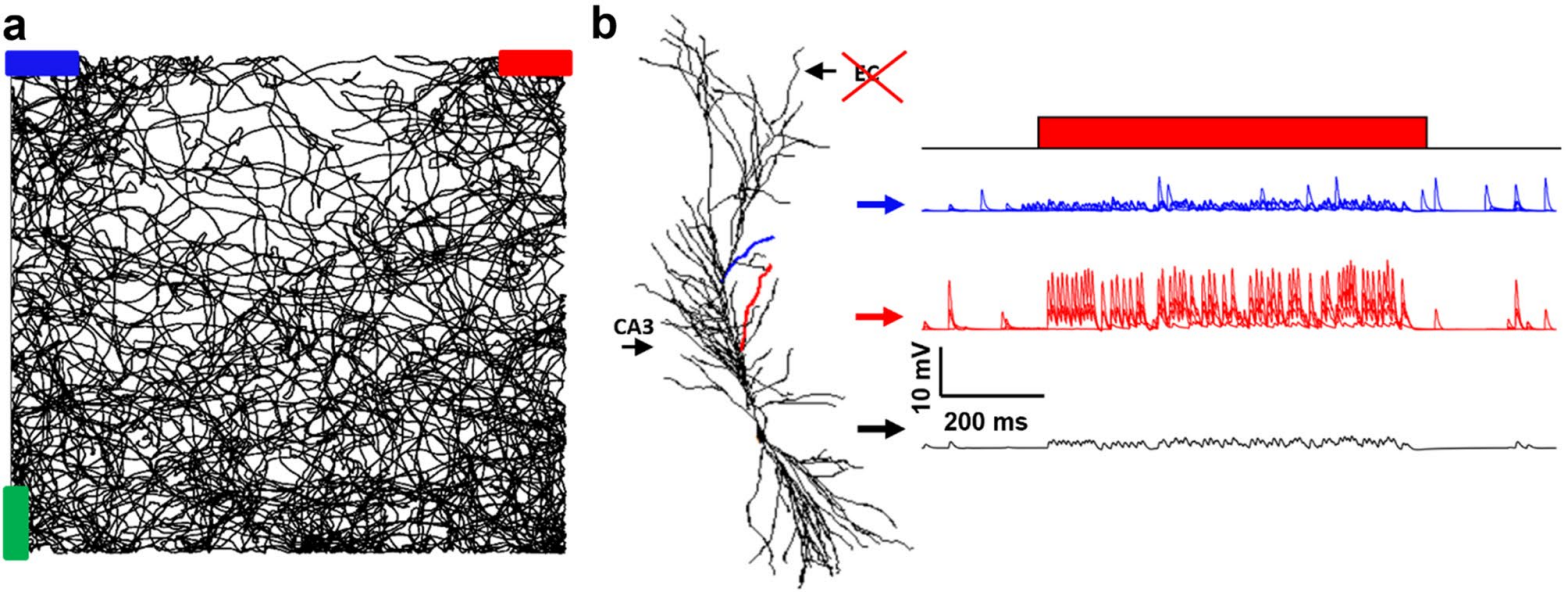
Simulation in the absence of the Plateau Potential. (**a**) Typical random trajectory followed by a virtual mouse during a simulation; (**b**) (left) the CA1 neuron used for all simulations; in blue is indicated a dendrite coding for the blue bar, in red a dendrite coding for the red bar; (right) membrane voltage in a dendrite coding for the blue bar (blue trace), in a dendrite coding for the red bar (in red), and in the soma (black), during a period in which the virtual mouse is looking at the red bar.

We first considered a typical simulation in which the virtual mouse explored a room with three objects (colored bars in Fig. 3a), without the Plateau Potential. The black line represents the animal’s random trajectory during the simulation. The recordings in Fig. 3b show the membrane potential at the soma (black trace) and at two dendritic locations (red and blue traces), corresponding to dendrites targeted by synapses that increase their activity in the presence of a red or blue object. Before the red object entered the visual field (red bar in Fig. 3b), the activity in both the blue and red dendrites was essentially random and at a slow (theta) frequency. As the red object entered the visual field, the synapses targeting the red dendrite (but not those targeting the blue dendrite) begun to be strongly (gamma) activated. However, this was not sufficient to elicit a dendritic or a somatic spike, or to activate STDP.

**Figure 4:**
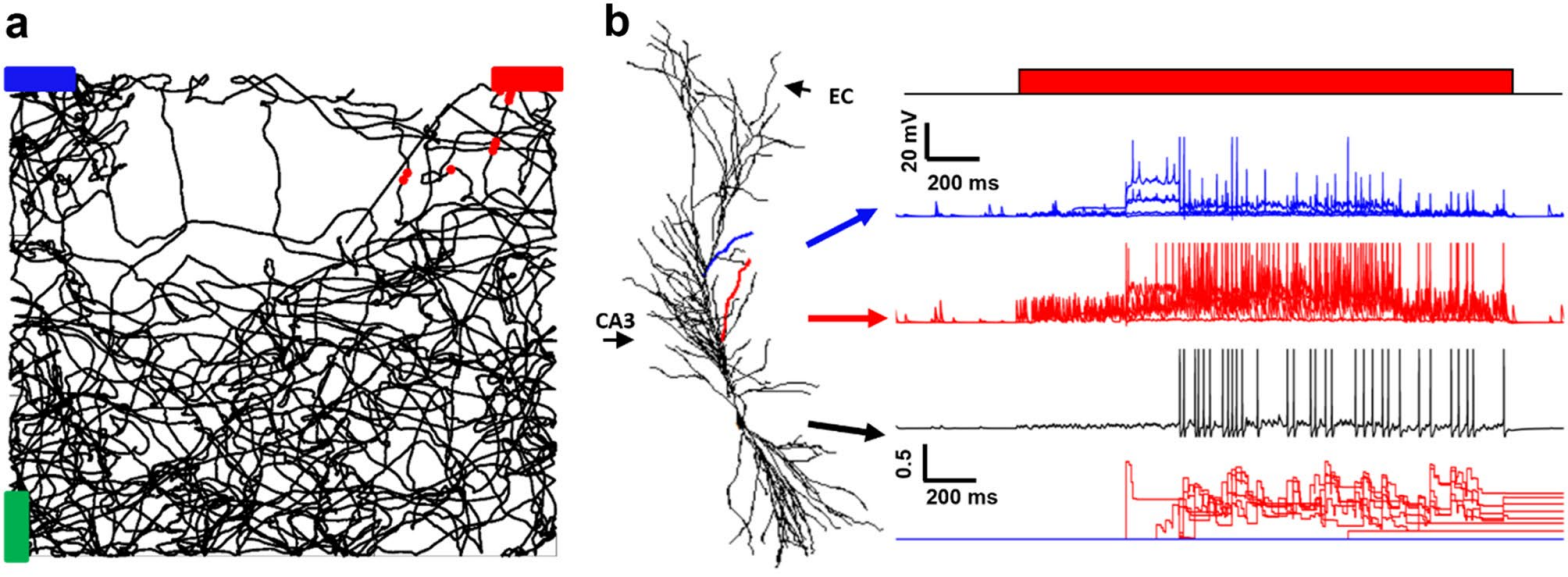
Typical simulation in the presence of the Plateau Potential. (**a**) The black line represents the virtual mouse trajectory while red dots indicate the positions where the neuron fires; (**b**) (left) the CA1 pyramidal neuron used for all simulations; blue indicates a dendrite coding for the blue bar, red a dendrite coding for the red bar; (right) membrane voltage at different locations (blue and red dendrites, and soma) while the mouse is looking at the red bar; the bottom plot represents the evolution of the synaptic weights.

In Fig. 4, we show the model results for the same simulation, but with the EC input activated when the red object was in the animal’s visual field, modeling a contextual/reward signal. The underlying assumption is that this input can contain information from an animal’s internal memories, and it can also be modulated by dopaminergic afferents carrying information on the reward^37^. The blue markers in Fig. 4a represent somatic spikes generated during the animal’s trajectory. As shown in Fig. 4b, under this condition the synapses on the red dendrite were strongly activated as soon as the red bar entered the visual field, as before, but this time the additional depolarization caused by the plateau potential generated dendritic spikes, which eventually propagated to the soma. This flurry of activity was sufficient to potentiate the synaptic weights on the red dendrites but not those on the blue ones (red and blue lines in the bottom plot of Fig. 4b). As the red bar disappeared from the visual field, the synaptic activity on the red dendrite fell back to background activity, but with potentiated synaptic weights: a Place Cell was formed.

It should be noted that Bittner et al.^38^ have convincingly shown that the time window of pre- and post-synaptic activity for inducing place-cell related plasticity can be up to several seconds wide. Our choice to use a relatively short timescale is again related to the computational tractability of full-scale models. A long time window can probably be related to the processes involved with intracellular calcium waves, which are known to propagate within a neuron at these time scales. However, the main concept in Bittner et al.^38^ is still based on the plasticity induced by the relative interval between (long lasting) pre- and post-synaptic activities, with plasticity induced proportionally to the overlap between them and maximally induced when they are coincident (see Fig. 3d in Bittner et. al.^38^). Our implementation can then be considered as a time compressed version, which allows our Place Cell to eventually (and quickly) respond to environmental cues on the fly during a simulation. This aspect is of a fundamental importance in implementing full-scale networks. In the future studies, it will be interesting to evaluate how cellular and network models perform when utilizing learning rules that operate at different timescales, employing rapid and slow timescale learning rules.

**Figure 5:**
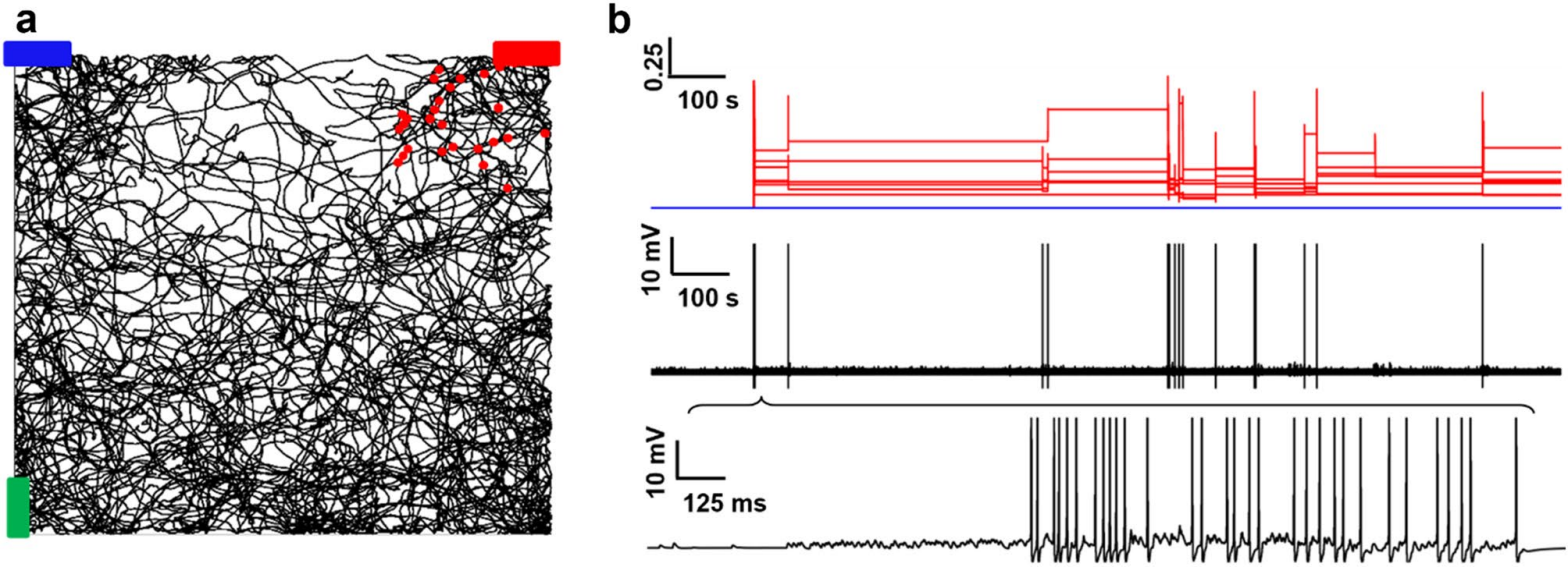
Place Cell formation and operation. (**a**) Typical mouse trajectory; The red dots indicate the positions where the neuron generated an action potential; (**b**) Synaptic weights evolution (top); Somatic membrane potential during an entire 10 min simulation (middle); the inset (bottom plot) shows 10 s of somatic potential during the activation of the plateau potential.

To test the robustness of Place Cell formation and its operation mechanism, we ran a simulation in which we let the virtual animal explore the room for approximately 15 min after the end of the EC input. In Fig. 5, we show the somatic activity and the time course of the synaptic weights (in the dendrites coding for the red object) during the entire simulation period (Fig. 5b top two graphs) and a 1 s window around the activation of the reward input (Fig. 5b, bottom plot). Note that the synaptic weights were left free to evolve, in contrast with most network implementations where the learning and recall phase are carried out independently, and the weights’ evolution is frozen during the recall phase. In our model, once the Place Cell is formed, through the integration of the proximal input coming from the CA3 region and the distal input coming from the EC, its operation remains stable during the subsequent exploration period. Even if the weights keep evolving with time, according to the cell’s activity history, their overall level stays strong enough to have somatic spikes during navigation, generated when the (virtual) animal enters a location associated with the red bar, consistently with what is observed experimentally^2,39^.

It should be clear that, being associated with a specific cue, the Place Cell activation in our model will be correlated with the specific spatial location of the object. This agrees with experimental findings showing that a PC will still fire if the room is rotated^41–43^ or if the cues spatial arrangement is modified^44^.

**Figure 6:**
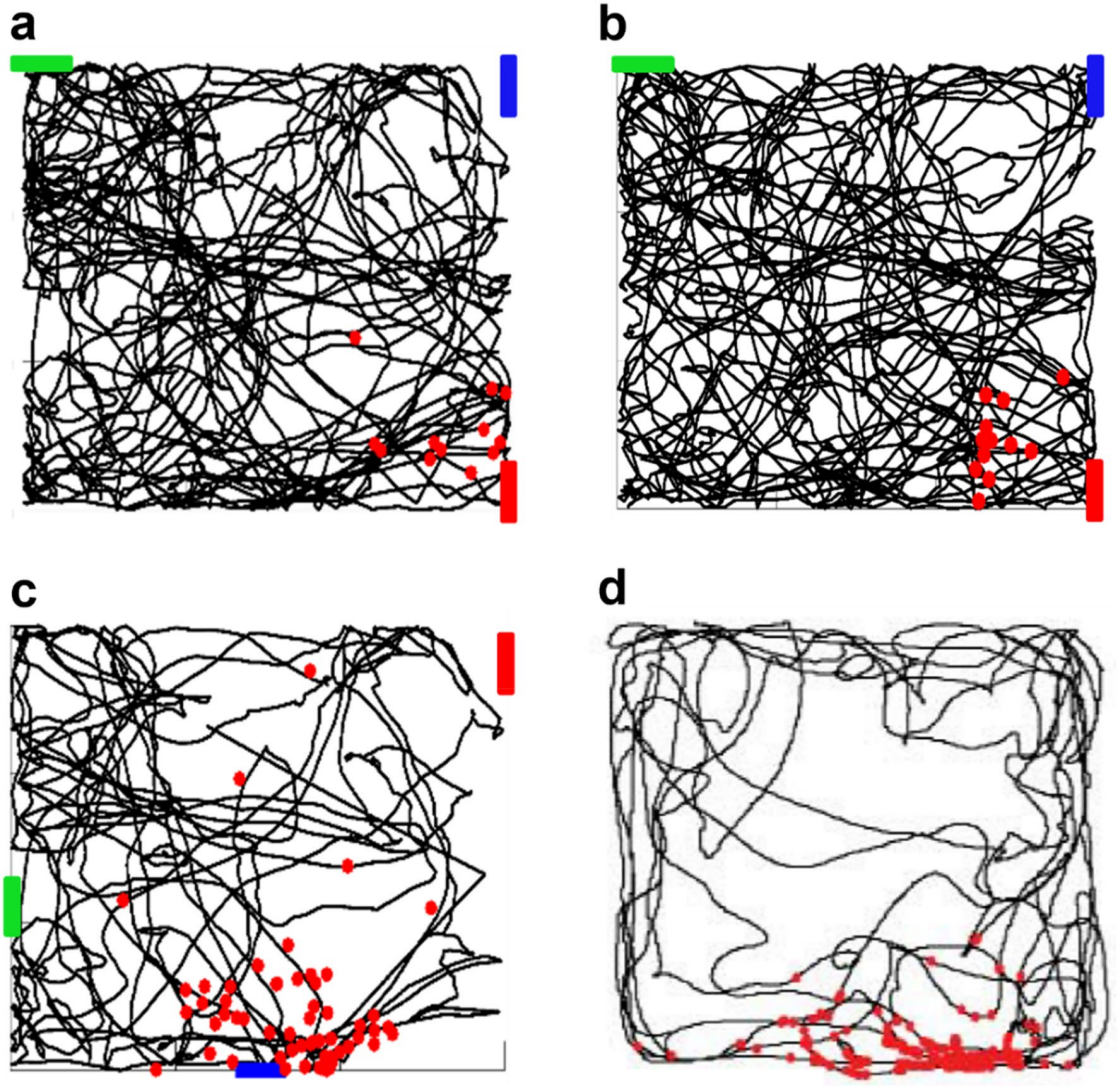
Model robustness and stability. (**a**) Rotated environment; (**b**) Different trajectory; (**c**) Different environment configuration; (**d**) Typical experimental results (Fig. 4a from Ref.^40^). The red dots represent the positions where the neuron generated action potentials.

To further test the robustness and flexibility of the model we carried out more simulations to see if the model neuron can still become a PC if: (a) the entire environment rotates, (b) the virtual mouse follows different trajectories, (c) the conformation of objects inside the arena is different. The red markers in Fig. 6 represent the spikes elicited during navigation. We first tested if the Place Cell remains stable when the environment is rotated; as we can see in Fig. 6a, the Place Cell’s activity (indicated by red markers) follows the rotation of the arena. Next, we validated the model by testing that the Place Cell can be also created when the virtual mouse followed a different random trajectory. As shown in Fig. 6b, this was confirmed by the model. Finally, in Fig. 6c we show a simulation in which the Place Cell was tuned with a different cue, using a different set of synapses (coding for a blue object). In this case, the Place Field was in another spatial position, as it often happens in experiments testing distinct rooms^44^. The PC was still well formed and operated as expected; this result demonstrates the robustness of the model and, implicitly, the flexibility for an individual neuron to encode different objects in its dendrites. The good qualitative agreement between our model and experimental findings is demonstrated by the plot shown in Fig. 6d, where we reported a typical experimental result^40^ obtained by recording a Place Cell activity and its firing when the animal is within a limited and specific area of the environment called place field.

**Figure 7:**
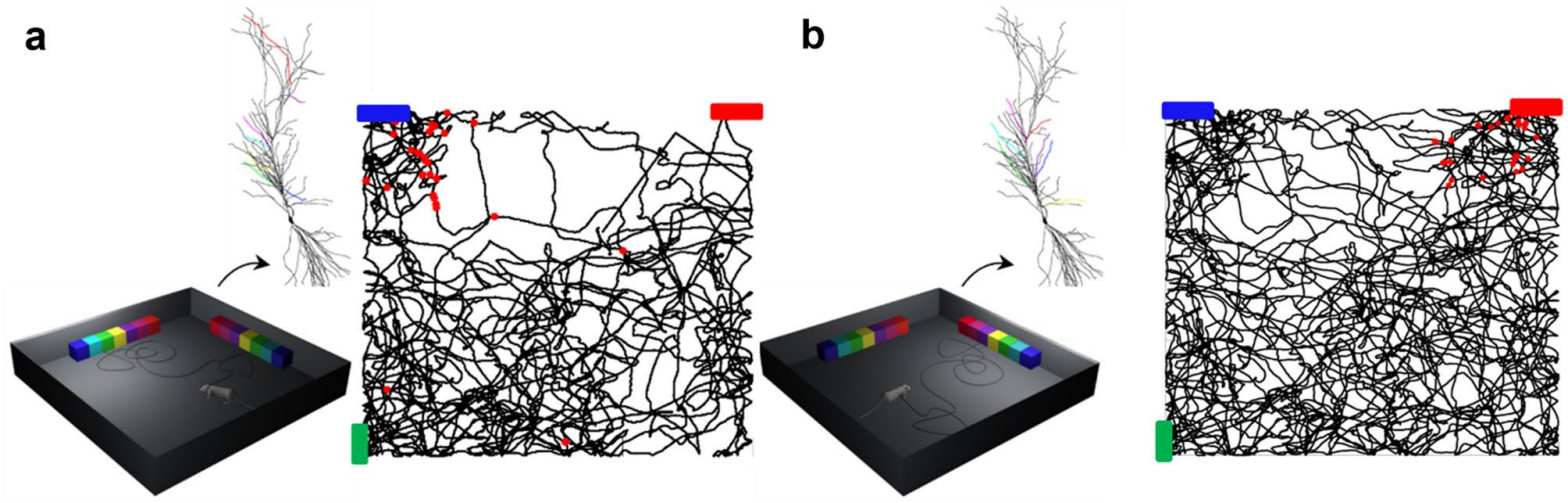
Place Cell formation in response to different object/place configuration. The reward signal was presented when the visual field included the blue (**a**) or the red (**b**) bar.

We also tested whether our model neuron could be tuned to a different object, thus creating a PC with a Place Field in a different position. In Fig. 7, we show the results when the PC was tuned with the blue bar (Fig. 7a), or with the red bar (Fig. 7b), using set of synapses located in different dendrites for each cue (see colored dendrites in Fig. 7a,b). In agreement with experimental findings^44^, within the same spatial context two different Place Cells can have two different place fields. In supplementary movie SM2, we show 2 simulations using the same trajectory but with the reward placed in different positions. The model thus suggests that the same neuron can represent different Place Cells when tuned to different objects. Taken together, these results demonstrate that the process of PC formation can be robustly supported by the conjunctive activation of the two main excitatory inputs on a CA1 pyramidal cell.

**Figure 8:**
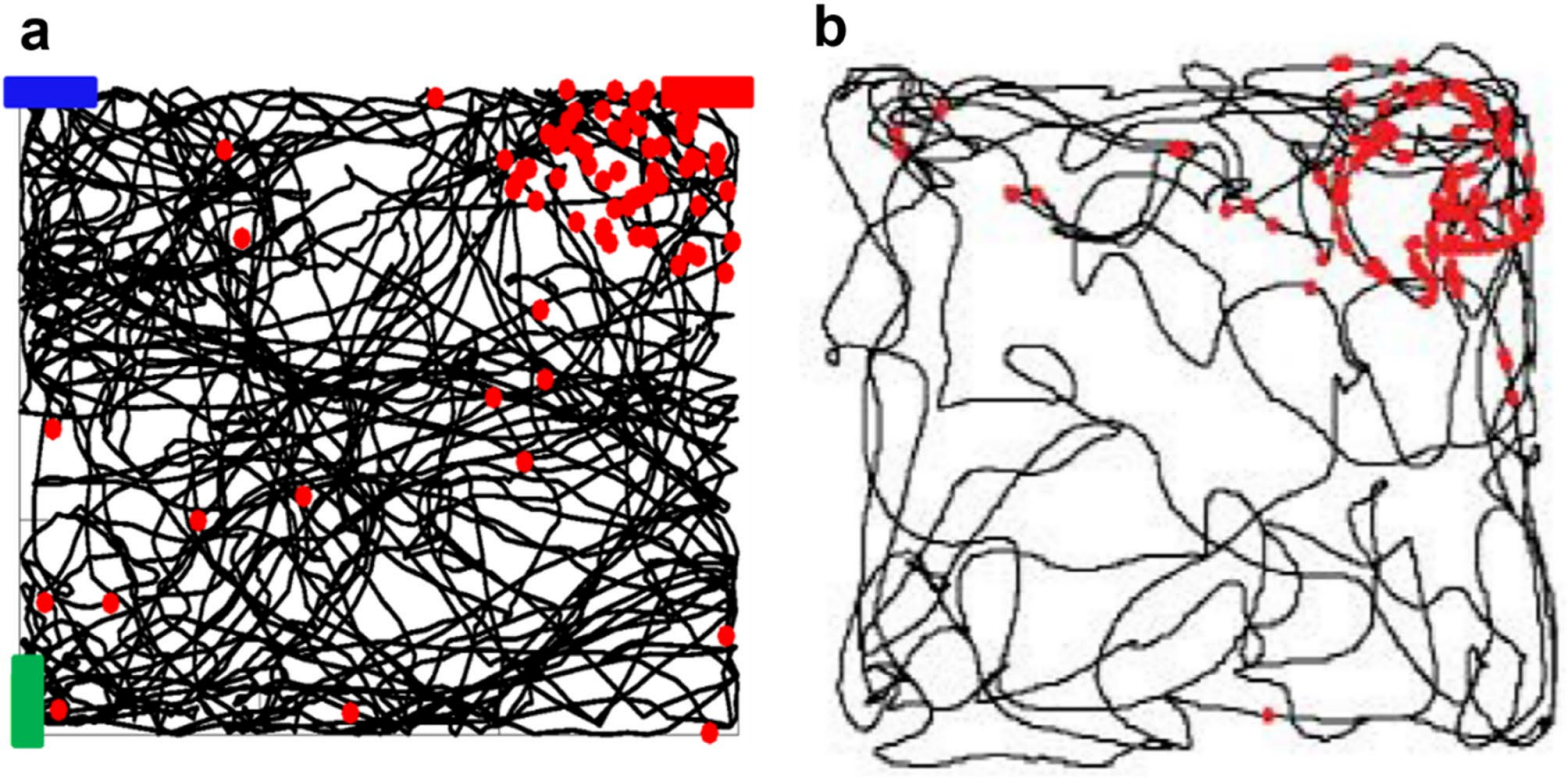
Comparison with experimental results. (**a**) The red markers represent spikes elicited by the neuron during 10 independent simulations after the training period; (**b**) Typical experimental results (Fig. 4a from Ref.40)^40^.

A more extensive demonstration of the robustness of PC formation and stability is illustrated in Fig. 8a, where we show the simulation findings from 10 different random trajectories, carried out after the training period, compared with another example of typical experimental results (Fig. 8b)^40^. Note that, in all cases, synaptic plasticity was not blocked, and the synapses were thus free to evolve according to the local dendritic activity.

## Discussion

Several neurophysiological and behavioral experiments have suggested that the hippocampus is essential for spatial navigation^1,2,39,45^, and that different cell types collaborate to create a cognitive map of the surrounding environment. The goal of this work was to introduce a computational model to study the cellular mechanisms underlying the formation of a Place Cell. This is important because a detailed implementation, at cellular level, of the mechanisms needed to turn a CA1 pyramidal neuron into a Place Cell, would allow to make more specific experimentally testable predictions on the mechanisms modulating cognitive functions and dysfunctions. In vivo and in vitro experimental findings suggested that the interaction between a distal input from the Entorhinal Cortex, and a proximal input from the CA3 area of the hippocampus, can induce an individual CA1 pyramidal neuron to function as a Place Cell^16^. Building upon these findings, the model suggested what could be the dendritic mechanisms underlying the formation of a Place Cell during navigation in an unknown environment. The key requirement emerging from our model is that environmental cues must be encoded in SCs inputs targeting a subset of oblique dendrites of a CA1 pyramidal neuron, which are activated above background activity when present in the animal’s field of view. In general, in a non-PC neuron, this increased synaptic activation is not sufficient to generate somatic action potentials. However, if this activation is associated with the sustained activation of a forward propagating distal input, as suggested by experiments, the overall local dendritic activity can generate synaptic plasticity selectively on those synapses coding for the input cues. This process leads to the formation of a PC that is now able to generate spikes every time that the same cue is present in the visual field, even if the environment is rotated, in agreement with experimental findings^41–43^.

A few computational models using or describing the formation of PCs have been published. However, in most cases, the process of PC formation is somewhat assumed ad hoc*.* Most computational models of PCs so far have been implemented using artificial neural networks, with two or more layers consisting of neuron-like elements^46,47^. Under these conditions, these type of models cannot consider or explain the binding process (which in a real neuron occurs through dendritic integration) needed to induce synaptic plasticity at the right time and dendritic location. For example, in Hartley et al.^48^ PCs were prewired to respond to the presence of any wall at a given distance and direction, firing when the input reached a threshold level. In Barry et al.^49^ the model was extended, adding experience-dependent plasticity to form Place Cells encoding the relative position to specific objects, through firing rates proportional to the weighted sum of their inputs. However, these PCs were implemented as simplified point-neurons. In the model discussed by Udakis et al.^50^, a PC was a pre-tuned, artificial, and rate-based two compartments neuron, in which excitatory and feedback/feedforward inhibitory inputs concur to stabilize its operation. A recent modeling paper^51^ suggested how a PC can be formed through the local interaction between a highly simplified principal neuron and an astrocyte, both activated by a given SCs input in the presence of a specific environment. Although this model is innovative and provides information on the role of astrocytes in the formation of Place Cells, it does not consider the conjunctive activation of the two spatially separated and independent signals that have been shown to be instrumental to create a Place Cell.

Our results suggest that the robustness and flexibility of the PC formation process relies on the spatiotemporal separation between the two proximal and distal inputs; inputs coding for different environmental cues are not potentiated during spatial exploration in the absence of a distally initiated plateau potential. The key point in our model is thus whether a forward propagating plateau potential is triggered or not. We assume that it could be activated by a relatively strong input in the tuft, using the general underlying assumption that this can be generated by inputs from the EC modulated by dopaminergic VTA afferents, which are known to exist^37^. This assumption is not in contradiction with Grienberger et al.^52^, where the relatively strong input can instead be most likely generated by amplification of a disinhibited EC input.

The timing of the reward input will thus determine which set of (active) synapses will be potentiated, and this allows for a given neuron to be tuned to place fields corresponding to one (or more) of the cues they encode in their oblique dendrites, without artificial supervision or ad hoc signals. Over time, maybe following synaptic turnover processes, the very same neuron could be tuned to encode different place fields, in response to different environmental cues. This is consistent with experimental evidence suggesting that CA1 pyramidal neurons receive inputs from presynaptic cells tuned to all possible spatial locations^16^.

A limitation of the model is the lack of a detailed implementation for NMDA-dependent calcium spikes, longitudinal calcium diffusion, and the modulatory action of intracellular calcium stores in the formation and forward propagation of dendritic calcium waves. In this version of the model, we have preferred to use an empirical but effective implementation, since a detailed implementation would have required at least one order of magnitude increase in simulation times, and would thus not be appropriate for large scale network implementation. We also decided not to include any feedback or feed-forward inhibition in the picture at this stage. The rationale for this decision was that, although inhibition has an important role in sculpting spatial coding in heterogeneous environments^52^, the simple and somewhat reduced model architecture used here as a proof of principle does not need it. Of course, it would be needed in a circuit or network implementation dealing with more complex environments, where its effect can be expected to significantly shape the formation of a Place Cell and, more generally, the formation of a place field. The model thus sits in the middle between the purely algorithmic ad hoc implementation almost universally used to model Place Cells and a more accurate biophysical implementation, such as that used in Bittner et al.^38^. The first type of implementation cannot give many physiologically useful insight or experimentally testable predictions, whereas the other type is computationally too expensive to be included in network simulations.

In conclusion, in this work we have introduced a model assuming control (i.e. healthy) conditions. It opens the way of making experimentally testable predictions on how hippocampus-related brain diseases can affect the formation of a Place Cell, since any pathological alterations/mutations of ion channel properties, synaptic transmission, or plasticity, can be straightforwardly implemented directly following experimental findings or suggestions. As we have previously demonstrated in a number of different cases^53–56^, the use of this type of model allows one to make direct assessment/predictions on how and to what extent specific channel mutations or modulations can be responsible for the disruption of a cognitive function, or how they can help in restoring normal functions during a pathological condition^35,57^. The model can thus be conveniently used, in isolation or in a detailed network, to support experimental suggestions on spatial navigation, predict pathological consequences, or suggest new treatments for brain diseases altering the intrinsic electrophysiological, morphological, or synaptic properties of a CA1 pyramidal neuron.

### Supplementary Information

Supplementary Video 1.

Supplementary Video 2.

Supplementary Figure S1.

## Data Availability

All the model and simulation data files are available on ModelDB (http://modeldb.yale.edu/267613).
