# Supplementary material for: A realistic computational model for the formation of a Place Cell
Source: Sci Rep. 2023 Dec 8;13:21763. doi: 10.1038/s41598-023-48183-5 (PMC10709575; doi:10.1038/s41598-023-48183-5)
Supplement: Supplementary file 3 — Supplementary Figure S1. [file 41598_2023_48183_MOESM3_ESM.docx]

**Supplementary material**


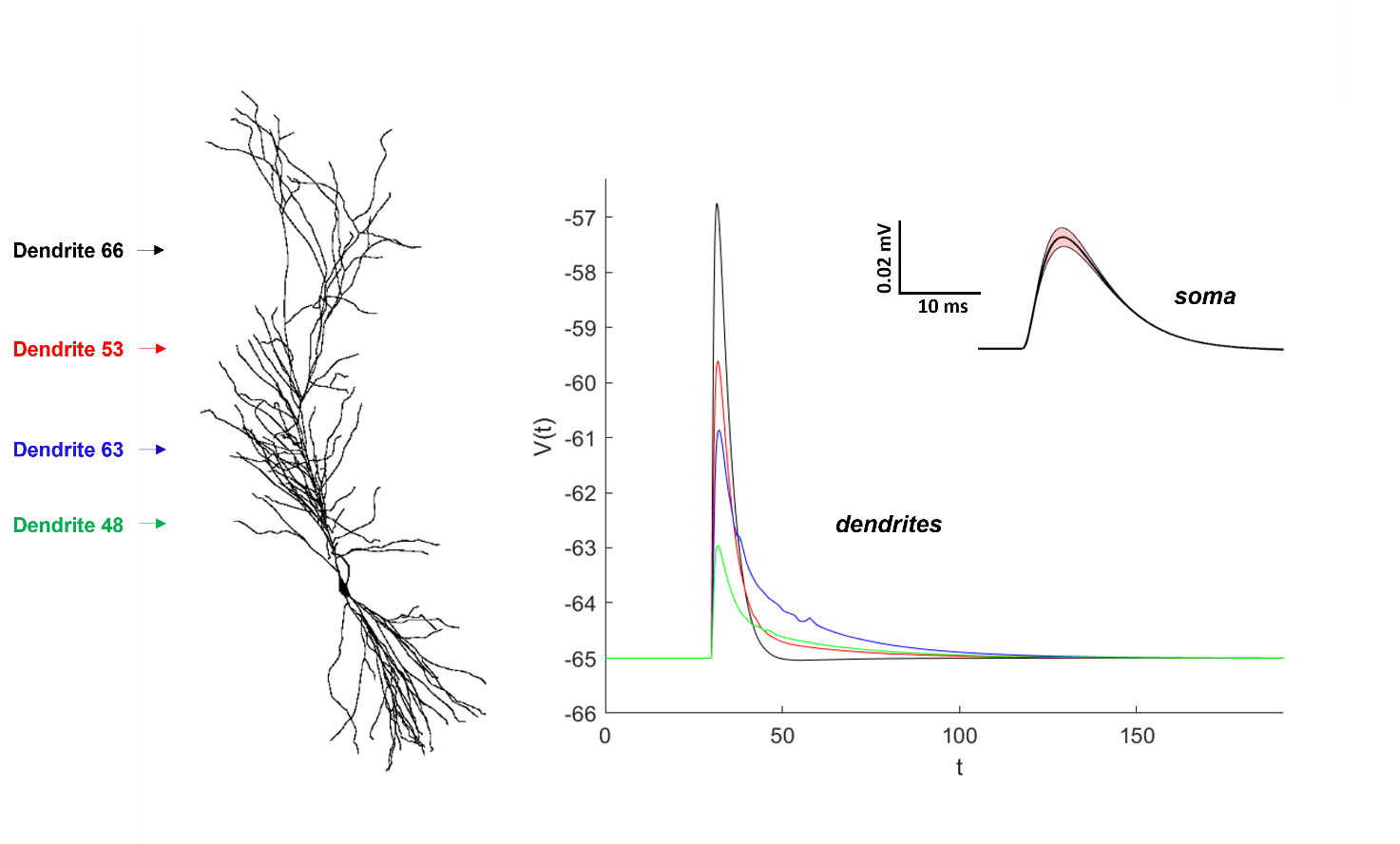


**Fig.S1**: Typical EPSPs recorded in four different oblique dendrites, with the inset showing the average somatic depolarization in response to individual synaptic activation (light red area represents variance).
